# The Effect of Solvothermal Conditions on the Properties of Three-Dimensional N-Doped Graphene Aerogels

**DOI:** 10.3390/nano9030350

**Published:** 2019-03-03

**Authors:** Alina Iuliana Pruna, Alfonso C. Cárcel, Adolfo Benedito, Enrique Giménez

**Affiliations:** 1 Universitat Politècnica de València (UPV), Instituto de Tecnología de Materiales, Camino de Vera s/n, 46022 Valencia, Spain; apruna@itm.upv.es (A.I.P.); enrique.gimenez@mcm.upv.es (E.G.); 2 Instituto Tecnológico del Plástico (AIMPLAS), 46980 Paterna (Valencia), Spain; abenedito@aimplas.es

**Keywords:** graphene oxide, ethylenediamine, aerogel, CO_2_ capture

## Abstract

Low-density three-dimensional (3D) N-doped graphene aerogels by a one-step solvothermal method in the presence of ethylenediamine (EDA) are reported. The gelation, formation, and properties of the aerogels were studied with solvothermal conditions, namely, operating temperature, time, graphene oxide (GO) concentration, and the GO/EDA w/w ratio. Two ranges of solvothermal conditions are employed: one involving an operating temperature below 100 °C and a conventional chemical reduction of GO with EDA at atmospheric pressure and a second one employing a higher temperature range up to 165 and a high pressure reduction with EDA. The results show that both solvothermal approaches allow for the fabrication of homogeneous N-doped 3D graphene aerogels with density values close to 10 mg cm^−3^. The measurements indicated that low values of GO concentration, temperature, and EDA are optimum for obtaining low-density 3D aerogels. N doping is improved with an EDA amount in lower temperature conditions. The N doping mechanism below 100 °C is dominated by the epoxy ring opening while at temperatures up to 165 °C both epoxy ring opening and amidation take place. The CO_2_ adsorption properties are strongly controlled by the nitrogen configuration, namely, pyridinic nitrogen in terms of its density.

## 1. Introduction

Strategies for mitigation of climate change due to anthropogenic sources are focused on reducing CO_2_ emissions from combustion of fossil fuels and on developing more selective and efficient CO_2_ capture systems [1]. 

Many carbon-based porous materials are appropriated for CO_2_ adsorption [2]. In particular, graphene-based three-dimensional (3D) porous aerogels obtained from reduced graphene oxide (rGO) exhibit very low density, a continuously interconnected porous network, and a large surface area, thanks to which they are considered one the most promising adsorbents for CO_2_ capture. In order to improve the CO_2_ capture capacity along with high CO_2_/N_2_ selectivity, rGO materials can be employed as composite materials with other adsorbent materials such as metal organic frameworks (MOFs) [3] or can incorporate radicals such as amine groups [4,5]. In addition, they are likely to facilitate the cyclic process of CO_2_ desorption during adsorbent regeneration by proper microwave heating [6]. Three-dimensional graphene networks exhibit interesting characteristics, including the presence of graphene oxide (GO) features in bulk volume, the improvement of practical applications in the field of adsorption by the easy separation of the adsorbent, and the prevention of a restacking of sheets in macro-scaled agglomerates during the adsorption process [7].

It is known that capture properties of aerogel adsorbents are controlled by the internal structure and porosity [8], the specific surface area, and the active sites, which in turn are strongly dependent on the synthesis route [9]. 

The solvothermal technique has been vastly researched as an already available and scalable method for the obtainment of GO aerogels from aqueous dispersions of GO sheets. In solvothermal treatment, GO sheets are reduced, resulting in the formation of a hydrogel that is converted into an aerogel upon freeze-drying. The reduction of GO and the subsequent formation of the hydrogel can be easily achieved by the effect of superheated water in the absence of a chemical reducing agent [10,11]. Recent literature [12,13] has shown that the temperature and duration of the hydrothermal process can be reduced when combining both hydrothermal effects and the chemical reduction by means of a reducing agent. Thus, improvement of the reduction degree and simultaneous gelation of rGO sheets can be achieved by adding varying reducing agents, such as ammonia, hydrazine, or vitamin C (L-ascorbic acid), to the GO dispersions [14,15].

In order to suppress the restacking of GO sheets during self-assembly, the functionalization of GO sheets by various molecules including ethylenediamine (EDA) was investigated [16,17,18]. However, the gelation of GO sheets functionalized by EDA has not yet been studied extensively and the fundamental roles behind gelation phenomena have not been clearly revealed either.

Nitrogen (N) doping was indicated as a viable approach to introduce active sites for various catalytic applications, where the selectivity and activity of N-doped graphene have been correlated to the N bonding configurations, e.g., pyridinic, pyrrolic, graphitic, or oxidized N. For example, the graphitic and edge-defect pyridinic N were reported to improve the oxygen reduction reaction [19,20]. Only recently, density functional theory calculations on N-doped graphene for the reduction of CO_2_ indicated that the highest active sites for conversion of CO_2_ are pyridinic N and graphitic N [21,22].

Here, we report a systematic study on GO gelation in the presence of EDA by a one-step solvothermal process [9]. EDA was used as a source of N doping in the rGO aerogels. In order to evaluate the gelation, formation, and properties of such N-doped rGO aerogels, varying parameters were employed so as to allow the evaluation of the effects of reduction and functionalization with the purpose of obtaining 3D N-doped aerogels with a combination of low density and good mechanical strength for CO_2_ capture. Therefore, the effects of gelation and functionalization were studied by a solvothermal approach based on functionalization of GO with EDA in a temperature range below 100 °C (LT), where reduction of GO by thermal treatment is minimized. On the other hand, a second solvothermal approach employing a temperature range of 120–165 °C (HT) considered the effects of both the reduction of GO and functionalization with EDA taking place simultaneously in the given temperature conditions. Moreover, the effects of a varying GO/EDA ratio, duration, and GO dispersion concentration are presented.

The obtained results show a marked effect of solvothermal synthesis conditions on the N defect configuration of N-doped rGO aerogels for improved CO_2_ capture performance.

## 2. Materials and Methods 

Aqueous GO slurry (Graphenea, 3 wt %) was employed as received. According to supplier data, the mean lateral particle size is 14.6 microns, and the monolayer content (measured in 0.5 mg·mL^−1^ solutions) is over 95%. Ethylenediamine (EDA) was purchased from Sigma-Aldrich (Madrid, Spain) and used as received. Aqueous GO dispersions ranging from 2 to 10 mg·mL^−1^ were used for obtaining the aerogels. Ethylenediamine was used in a GO/EDA w/w ratio ranging from 1:0 to 1:6. The volume and density of the hydro/aerogels were controlled by the size of the vial. The corresponding amount of EDA was added to a vial containing 15 mL of GO dispersion. The mixtures were then subjected to either LT or HT routes for the synthesis of rGO hydrogels. The LT route was carried out by keeping the mixture at 85 °C. In the case of HT route, the mixed solution was introduced into a Teflon-lined autoclave and heated at different temperatures (120, 140, and 165 °C) for varying duration, ranging 6–24 h. The resulting functionalized GO hydrogels were washed with water and converted into graphene aerogels by freeze-drying at −80 °C under a high vacuum at 0.05 mbar (Telstar Lyo Quest freeze-dryer, three-directional cooling, a cooling rate of about 12 degrees min^−1^, according to manufacturer, sublimation at 20° for 48 h at 0.015 mbar).

The surface morphologies of rGO aerogels were analyzed by scanning electron microscopy (SEM) (JSM 6300, JEOL, Tokyo, Japan) equipped with energy dispersive X-ray spectroscopy (EDS, Oxford Instruments, Bristol, UK). EDS analysis was employed in order to assess the N content in the obtained aerogels. The apparent density of the aerogels was calculated by dividing the weight to their volume. X-ray photoelectron spectroscopy (XPS) analysis of GO and functionalized rGO aerogels was performed with a photoelectron spectrometer VG-Microtech Multilab 3000 (Thermo Fisher Scientific Inc., Waltham, USA). Raman spectra were measured by irradiation with laser light at 532 nm with an Xplora spectrometer (Horiba, Villeneuve d´Ascq, France).

Nitrogen and CO_2_ adsorption/desorption isotherms were conducted using a ASAP 2420 analyzer (Micromeritics, Norcross, GA, USA). The Brunauer–Emmett–Teller (BET) method was used to calculate the specific surface areas of dried samples at 77 K. The CO_2_ adsorption isotherms of the aerogels were estimated at 298 K, up to a 1 bar pressure region. The samples were outgassed under vacuum at 80 °C for 24 h before adsorption measurement.

## 3. Results

In order to study the gelation mechanism and effects on formation of aerogel, a varying GO/EDA w/w ratio ranging from 1:0 to 1:6 was employed. The critical gel concentration of GO was another parameter to consider, e.g., gelation was reported to occur at values as low as 0.075–0.125 mg·mL^−1^, although the aerogels obtained at such low values did not exhibit good mechanical strength [23]. In this work, GO concentration was varied from 2 to 10 mg·mL^−1^.

**Figure 1:**
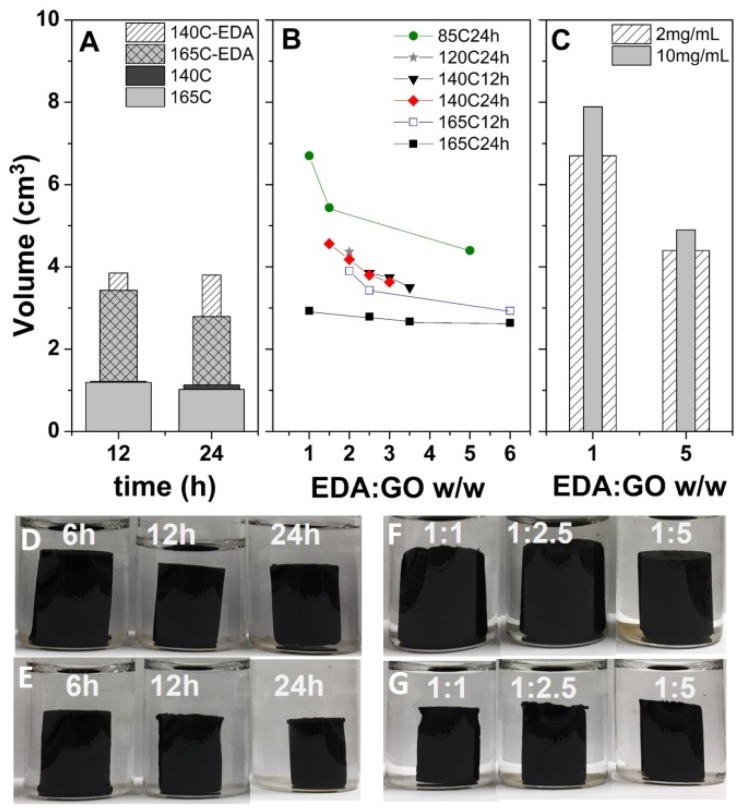
Hydrogel volume evolution in the absence and in the presence of ethylenediamine (EDA) (2.5) (**A**), evolution with EDA content (**B**), and evolution with graphene oxide (GO) concentration at 85 °C for 24 h (**C**). Digital images of 1:2.5 GO/EDA hydrogel evolution with time at 140 and 165 °C (**D**,**E**). Digital images of hydrogel evolution with EDA at 85 and 165 °C for 24 h (**F**,**G**).

The volume of the rGO hydrogel obtained by the HT route in the absence and in the presence of a given GO/EDA w/w ratio (1:2.5) is depicted in Figure 1A. One can observe volume values reaching 1.1−1.2 cm^3^ for the non-modified hydrogel. Moreover, only a slight decrease in volume was observed with temperature and time in the absence of EDA. Upon functionalization with EDA, the corresponding hydrogels increased in volume as depicted in Figure 1A. It is suggested that the amine groups from EDA act as a swelling agent, preventing GO sheets from self-stacking during the reduction and gelation process. The hydrogel compaction increases with temperature and time, as confirmed by the digital images in Figure 1D,E, depicting the hydrogel volume evolution with time at 140 and 165 °C, respectively.

The functionalization degree was investigated by recording the evolution of hydrogel volume with the GO/EDA w/w ratio, as depicted in Figure 1B. A decreasing trend of the hydrogel volume with EDA content can be observed for all temperature and duration conditions, as confirmed by digital images in Figure 1F,G, depicting the hydrogel volume evolution with GO/EDA w/w at 85 and 165 °C, respectively. The highest temperature and process duration result in the lowest volume values. By analyzing the slopes of volume evolution with EDA and temperatures, a convergence to a GO/EDA ratio of 1:2.5 was observed. On the other hand, the volume evolution of N-doped hydrogels depicted in Figure 1C shows increased hydrogel volume with the concentration of GO in initial dispersion. 

**Figure 2:**
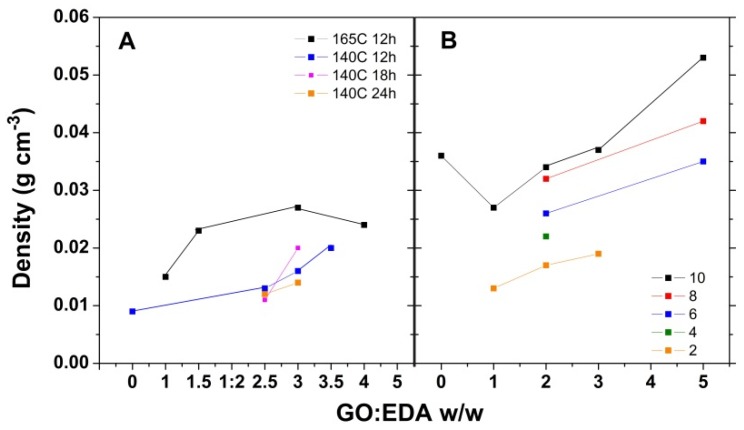
Aerogel density evolution by the HT approach (**A**) and LT one (**B**) as a function of GO/EDA ratio for varying GO concentration (mg·mL^−1^).

The density values for the aerogels obtained by the solvothermal method as a function of temperature, EDA content, and GO concentration are depicted in Figure 2. The results indicate that the aerogel density greatly depends on the employed process conditions, and they are in high agreement with the gelation ones. Both approaches result in low-density aerogels at lower EDA content. The density evolution with GO concentration depicted in Figure 2B indicates that a low concentration of GO in the initial dispersion is recommended for obtaining low-density aerogels.

**Figure 3:**
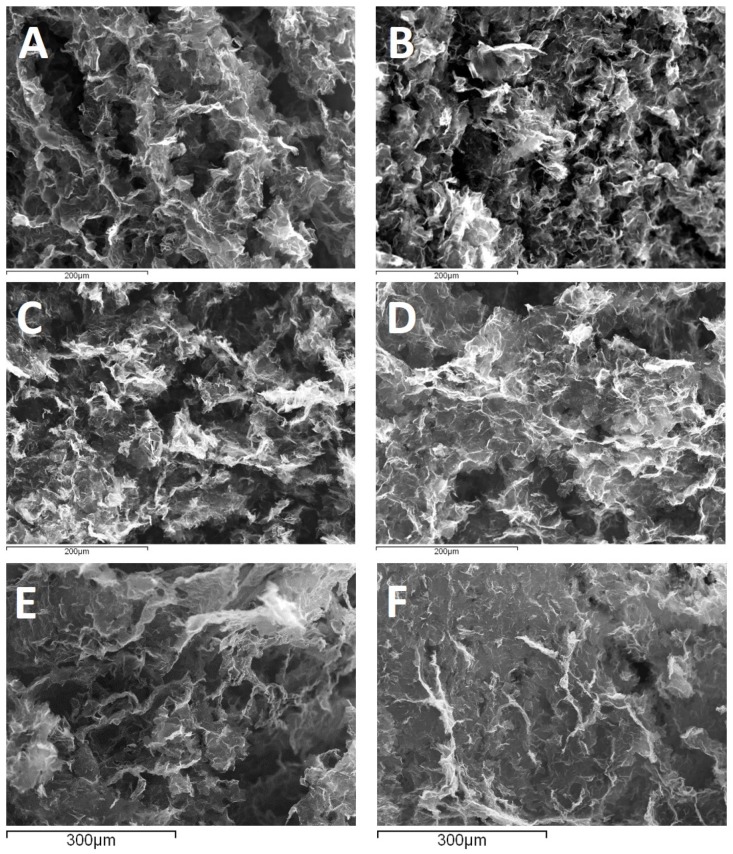
SEM images of EDA-functionalized GO aerogels. At 165 °C, 12 h, a 2 mg·mL^−1^ GO dispersion where GO/EDA = 1:2.5 (**A**) and 1:6 (**B**). From a 2 mg·mL^−1^ GO dispersion at 140 °C where GO/EDA = 1:2.5 for 12 h (**C**) and 18 h (**D**). From a 2 mg·mL^−1^ GO dispersion (**E**) and a 10 mg·mL^−1^ dispersion (**F**) where GO/EDA = 1:2.5 at 85 °C.

The compaction and porosity of obtained aerogels were further investigated by SEM measurements. As can be observed in Figure 3, the internal structure of aerogels is similar for both approaches, showing curved and wrinkled sheets of stacked GO flakes. The SEM images in Figure 3 show an increased stacking of sheets with temperature, EDA content, solvothermal process duration, and GO concentration in initial dispersion volume in agreement with gelation and density results.

**Figure 4:**
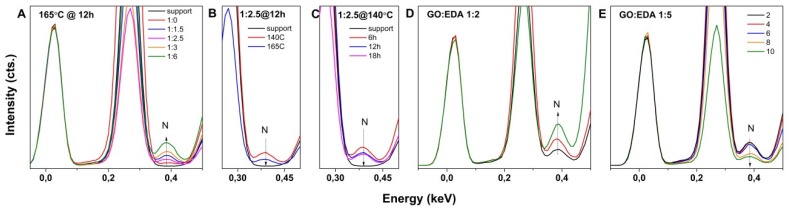
EDS spectra of EDA-functionalized GO aerogels from a 2 mg·mL^−1^ GO dispersion by the HT approach (**A**–**C**) and the LT approach (**D**,**E**) as a function of the GO/EDA ratio, temperature, duration, and GO concentration (mg·mL^−1^).

EDS spectroscopy results indicated that solvothermal approach is suitable for incorporating nitrogen into the aerogels. The nitrogen content was shown to increase with the amount of EDA as seen in Figure 4A, depicting the evolution of nitrogen EDS peak intensity for the aerogels obtained by the HT approach at 165 °C, which is in agreement with previous results of an increasing functionalization degree with EDA addition. However, the increase in operating temperature and solvothermal process duration negatively impact the nitrogen content, as shown in Figure 4B,C, which is attributed to amine loss [24]. Figure 4D,E show the evolution of EDS spectra with GO concentration in the initial volume of dispersion for a GO/EDA ratio of 1:2 (D) and a GO/EDA ratio of 1:5 (E) by the LT approach. The nitrogen content in the obtained aerogels was observed to increase with GO concentration at low EDA but decrease at higher EDA.

**Figure 5:**
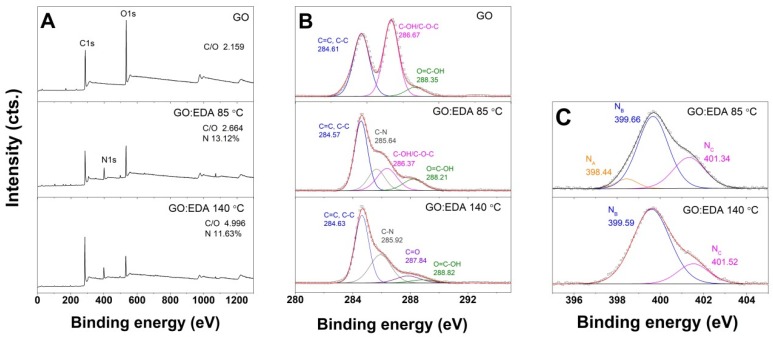
XPS spectra for EDA-functionalized GO aerogels obtained by the two treatment routes, at 85 °C (LT) and 140 °C (HT), respectively, from a 2 mg·mL^−1^ GO dispersion and ratio GO/EDA = 1:2.5: survey (**A**), high resolution C1s (**B**) and high resolution N1s (**C**).

XPS analysis was further carried out in order to investigate the functionalization with EDA of the GO aerogels by a one-step solvothermal approach. For exemplification, the survey spectra of the original GO and the aerogels obtained by varying the solvothermal approach (HT and LT) at the same GO/EDA ratio of 1:2.5 for a 2 mg·mL^−1^ GO dispersion are shown in Figure 5A. The N1s peak appears around 400 eV in the XPS survey spectra of modified aerogels and its content reaches 13.12% (LT) and 11.63% (HT). Besides the nitrogen content variation in the obtained aerogels, a higher reduction degree of the GO sheets was observed at a higher operating temperature, as can be readily observed by the marked increase in the C/O ratio in Figure 5 from 2.664 (LT) to 4.996 (HT). Residual oxygen content in rGO aerogels is clearly higher for the low temperature process (23.7% for LT vs. 14.81% for HT route). These results are in good agreement with the hydrogel volume contraction and aerogel density when process temperature increases. A further insight into the possible reaction mechanisms of EDA with the oxygen groups in GO by the two approaches is presented in the following section by recording the high resolution C1s and N1s spectra of the original GO vs. the modified rGO sheets in the obtained aerogels depicted in Figure 5B,C.

The C1s spectra for the GO starting material is deconvoluted into three components located at 284.61, 286.67, and 288.35 eV which are assigned to C=C/C–C, C–OH/C–O–C, and COOH, respectively. The functionalization with EDA simultaneously with the formation of the rGO aerogels is clearly influenced by the solvothermal conditions, as observed by the deconvolution of the C1s spectra into four fitting curves but with different assignments: in the case of the LT approach, the deconvolution of C1s spectra includes, besides the original GO, a fitting curve with the binding energy located at 285.64 eV, which is attributed to a C–N bond in the amine [25], while the spectra recorded for the aerogel obtained by the HT approach is deconvoluted into four fitting curves with binding energies located at 284.63, 285.92, 287.84, and 288.82 eV, which are assigned to C=C/C–C and C–N bonds in the amine, C=O, and COOH, respectively.

The N1s spectra in Figure 5c were deconvoluted into three fitting curves with binding energies located at 398.44, 399.66, and 401.34 eV, assigned to pyridinic CH_2_–NH_2_, pyrrolic or amine moieties N(–NH), and graphitic C–N=C for the LT case and two curves centered at 399.55 and 401.5 eV for the HT approach [26,27,28].

**Figure 6:**
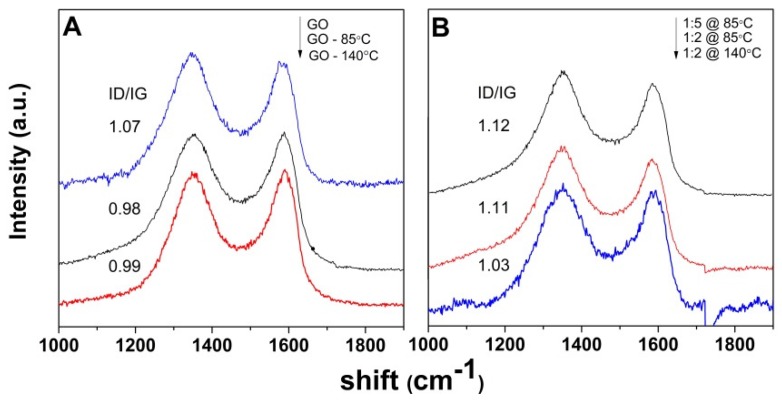
Raman spectra of GO and GO aerogels obtained at 85 and 140 °C (**A**) and EDA-functionalized GO aerogels from a 2 mg·mL^−1^ GO dispersion and varying GO/EDA ratio (**B**).

Raman spectroscopy measurements were further carried out in order to investigate the effect of both the functionalization and reduction of the defect content degree in GO aerogels. Typical peaks in the Raman spectra of GO depicted in Figure 6A are the G band at 1593 cm^−1^, associated with the aromatic sp^2^ network of graphite, and the D band at 1352 cm^−1^, associated with disorder originating from defects such as vacancies, grain boundaries, or amorphous carbon species [29]. 

The peak intensity ratio ID/IG is thus a good indicator of the existing surface defects. The spectra in Figure 6 show the evolution of ID/IG of N-doped GO aerogels with respect to the non-functionalized counterparts. While the ID/IG ratio shows a discrete decrease with the reduction of GO in the absence of EDA for both solvothermal approaches (Figure 6A), the N-doped counterparts exhibit an increased ID/IG ratio irrespectively of the solvothermal approach (Figure 6B).

Applicability of the obtained GO aerogels for CO_2_ capture was investigated by adsorption isotherm measurements. In this respect, the reactive surface was measured by nitrogen adsorption isotherms with the BET (Brunauer–Emmett–Teller) method at 77 K. The BET surface area of the original freeze-dried GO powder was determined as only 10.7 m^2^·g, similarly to other freeze-dried aerogels [30]. On the other hand, the N-doped graphene aerogels prepared in the optimum conditions by both LT and HT routes, namely, a 2 mg·mL^−1^ GO dispersion, where GO/EDA = 1:2.5 at 85 and 140 °C, respectively, showed a BET surface of 20 and 156 m^2^·g^−1^, respectively.

**Figure 7:**
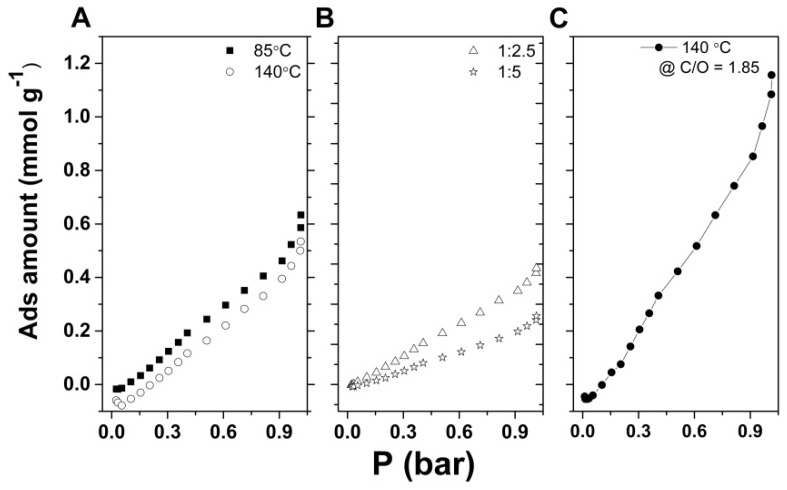
CO_2_ adsorption isotherm for EDA-functionalized GO aerogels obtained from a 2 mg·mL^−1^ GO dispersion where GO/EDA = 1:2.5 at 85 and 140 °C (**A**), aerogels obtained from a 6 mg·mL^−1^ GO dispersion where GO/EDA = 1:2 and 1:5 (**B**), and aerogel obtained at 140 °C from a 2 mg·mL^−1^ dispersion of GO where C/O = 1.85 and GO/EDA = 1:2.5 (**C**).

The performance of EDA-functionalized rGO aerogels for CO_2_ adsorption was tested at 298 K, and the values were recorded up to 1 bar. As can be observed in Figure 7A, both types of aerogels follow a similar trend, reaching values around 0.5–0.6 mmol·g^−1^ CO_2_ at 1 bar, which is in line with the reported literature for other carbonaceous materials [31]. The aerogels obtained from a 2 mg·mL^−1^ GO dispersion by the LT approach exhibit improved CO_2_ adsorption properties with respect to the one obtained by the HT approach (Figure 7A). 

The capture properties of modified aerogels obtained from more concentrated GO dispersions (6 mg·mL^−1^), thus having more oxygen groups in the same aerogel volume, were determined, as shown in Figure 7B. However, a decrease in CO_2_ adsorption capacity was observed, and increasing the GO/EDA ratio did not improve this adsorption. On the other hand, the modified aerogel in Figure 7C shows an improvement in CO_2_ adsorption properties with increased oxygen content of starting GO.

**Figure 8:**
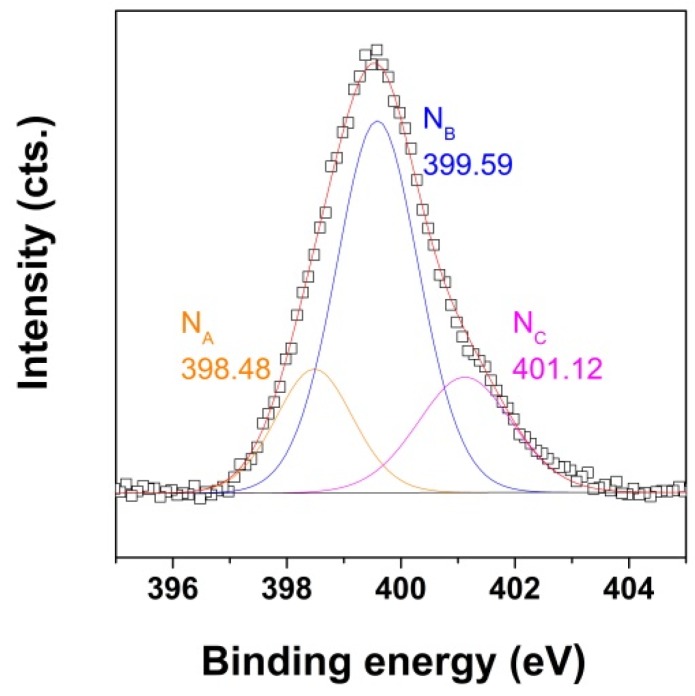
XPS N1s spectra for EDA-functionalized GO aerogels obtained aerogel obtained at 140 °C from a 2 mg·mL^−1^ dispersion of GO where C/O = 1.85 and GO/EDA = 1:2.5.

In order to confirm the effect of oxidation degree on the capture property of the N-doped aerogel, N1s spectra of the corresponding aerogel (Figure 7**C**) were recorded, as depicted in Figure 8. The quantification of the N configurations with the initial GO C/O ratio and synthesis conditions is presented in Table 1. 

## 4. Discussion

It is known that, in a GO suspension, the individual GO sheets are subject to electrostatic repulsion from the functional groups on GO sheets and to van der Waals interaction between the basal planes [32]. Two processes take place simultaneously in the solvothermal process for graphene aerogels: the removal of oxygen groups in GO (reduction) due to thermal effects and functionalization with EDA molecules. 

The formation of hydrogels was first evaluated from the apparent volume of the hydrogel obtained in given conditions. While no monolith could be formed at 85 °C, the formation of hydrogel monoliths at higher temperature (HT route) indicates that the self-assembly of rGO hydrogel is induced by the chemical reduction of GO. The volume of EDA-modified hydrogels increases irrespectively of solvothermal conditions confirming the successful attachment of amine moieties on the graphene flakes, while the larger volume gradient with temperature with respect to non-modified hydrogels (Figure 1A) indicates simultaneous reduction and functionalization taking place by the HT route. The lowest volume values were observed at the highest temperature and duration as depicted in Figure 1B and are attributed to increased removal of oxygen-functional groups of GO and decoration with amine moieties. However, the instability of amine moieties may also account for the decrease in volume at high temperature, as suggested elsewhere [24]. The volume gradient appears to be markedly affected as the temperature decreases to 85 °C, which indicates that the reduction kinetics is markedly decreased with respect to functionalization at temperatures below 100 °C. The volume convergence to a GO/EDA ratio of 1:2.5 suggests that such a ratio is optimal for effective functionalization with EDA by the solvothermal process. Increasing the concentration of initial GO dispersions results in a hydrogel volume increase due to an increased number of GO sheets in the same confined reaction volume. With regard to the density of the obtained aerogels by the HT route, it was observed the optimum GO/EDA ratio was up to 1:2.5 and a low GO concentration, namely, 2 mg·mL^−1^, in agreement with the gelation results. 

The lower density of the aerogels achieved by the HT route with respect to the LT route from a 2 mg·mL^−1^ GO dispersion is attributed to a loss of oxygen groups at an operating temperature that could otherwise be functionalized at lower temperature. 

The increase in nitrogen content with EDA as observed by EDS measurements indicates an improvement in the degree of functionalization. Considering the removal of oxygen groups negligible by the LT approach, the evolution of nitrogen content with EDA content and GO concentration is attributed to the impeded penetration of EDA molecules between the GO sheets towards functionalization due to the agglomeration of the increased number of GO sheets in the same confined volume. An increase in nitrogen content was observed at lower GO concentrations as the GO sheets were readily available for functionalization. 

The appearance of the N1s peak in the XPS survey spectra for the modified aerogels with respect to the original GO nanomaterial is attributed to the amine loading and confirms the successful incorporation of these moieties by the solvothermal approach. The lower nitrogen content in the aerogels obtained at higher temperature (HT) agrees with the gelation results. Although the reaction between GO and amine functions has been indicated to take place via the ring opening of the epoxides, rather than by amidation [33,34,35], several derivatization reactions may occur concomitantly due to the high reactivity of the oxygenated groups in GO, namely, epoxy, hydroxyl and carboxyl groups. Thus, the disappearance of the peak at 286.67 eV of the starting GO material and the appearance of the small C=O peak in the functionalized GO aerogel obtained by the HT approach indicate a transformation of epoxide to carbonyl [36]. The evolution of C–OH/C–O–C and COOH components in the LT route indicates a predominant epoxy ring opening reaction [37,38], while in the HT case the functionalization of GO with EDA is associated with the epoxy ring opening simultaneously with amidation [39]. The N1s spectra in Figure 5C confirm the difference in functionalization by the LT and HT approaches: while the pyrrolic N1s has the major contribution in both cases, reaching 64.9 at % and 81.5 at % (from total N content), respectively, there is a different evolution in the other nitrogen configurations, resulting in an area ratio of 0.1:1:0.44 and 1:0.23 for the LT and HT route, respectively. 

The successful loading of amine moieties on the surface of GO sheets was confirmed as well by the increased degree of defects in the modified aerogels, as indicated by the enhanced ID/IG ratio in the Raman spectra in Figure 6. Improvement of the functionalization degree by the LT approach and with the EDA amount is indicated by the higher ID/IG ratio, as depicted in Figure 6B, in agreement with EDS and XPS results. 

It was reported that the accessible surface area and framework stability is markedly dependent on the type and length of cross-linker used (length of the pillaring unit) as well as synthesis temperature [40]. The low values for the BET surface area could be explained by the degassing of aerogels at low temperature employed in order to avoid the reduction of GO and loss of amine moieties (80 °C) and the existence of ultra-micropores for which the apparent BET value is smaller than reality [41]. 

Since the apparent BET surface area alone cannot support the capture results in Figure 7A, other factors must be considered. Amine content adjustments by controlling the GO concentration, the EDA content, or the operating temperature were studied. The less reduced hydrogels containing more nitrogen obtained by the LT approach show a slightly improved adsorption ability. Thus, the residual oxygen functional groups in LT aerogels were supposed to aid in enhancing the physical CO_2_ adsorption capacity because they contain an electron-donating oxygen atom that could participate in electrostatic interactions with CO_2_, thus increasing capture ability [42], but the capture properties are negatively affected by GO concentration or further EDA addition (Figure 7B), which indicates that the compaction and higher density of the aerogels are detrimental to CO_2_ capture. On the other hand, adjusting the amine content by improving the functionalization degree in the aerogel through the C/O ratio of GO (e.g., increasing oxygen content to 1.85 with respect to 2.159 of the original GO) was observed to greatly enhance CO_2_ capture (Figure 7C), which indicates that the density of introduced amine moieties per GO sheet is of paramount importance for enhancing capture properties. 

As can be observed in Figure 8 and Table 1, increasing the oxidation degree in GO results in pyridinic, pyrrolic, and graphitic N configurations that are similar to the aerogels obtained from a lower oxidation degree GO but by the LT approach, as previously depicted in Figure 5C. The intensity for pyridinic N is higher in this case. A recent study indicated that CO_2_ adsorption on N-induced defects in graphene is unfavored for pyrrolic N at 300 K, while adsorption at graphitic N is slightly favored [43]. Taking into account the increase in CO_2_ capture with the increase of the pyridinic N contribution, as an effect of improving the oxidation degree in an initial GO nanomaterial, it is suggested that improvement of the adsorption property is strongly dependent on N configuration, namely, pyridinic N, in agreement with previous studies [21]. 

Regarding the adsorption mechanism, it is known that CO_2_ interactions with N-doped graphene surfaces are either dominated by physisorption at moderate-to-high pressures or by chemisorption at lower pressures [44]. The CO_2_ capture behavior seems to confirm that adsorption of CO_2_ at low pressure on the EDA-modified rGO aerogels is mainly due to chemisorption capture mechanisms promoted by the existence of the introduced N defects [45]. The obtained results indicate that the CO_2_ adsorption mechanism of the N-doped rGO aerogels is complex and greatly depends on a suitable balance between nitrogen configuration, amine stability, and porosity properties induced by the rGO sheets.

## 5. Conclusions

The fabrication of N-doped rGO aerogels by a one-step solvothermal approach involving simultaneous aqueous reduction and functionalization with EDA is reported. The results indicated HT approach for increasing GO reduction level, while LT approach improves functionalization degree. Low-density aerogels can be achieved from a 2 mg·mL^−1^ GO dispersion at low operating temperature and low GO/EDA w/w ratio. XPS results indicated functionalization mechanism by the LT approach takes place by epoxy ring opening while in the HT case epoxy ring opening simultaneous with amidation is observed. The CO_2_ adsorption properties were found to be strongly controlled by pyridinic N configuration in the doped rGO aerogels and not simply related to BET surface area. Chemical sorption mechanism is suggested as the main contributor to the CO_2_ capture at low pressures. Further tailoring of the N configuration, stability and aerogel porosity and reduction degree is advised for adsorption enhancement.

## Figures and Tables

**Table 1:** N configuration (XPS quantification) of N-doped graphene aerogels as a function of synthesis conditions (temperature, GO/EDA, and GO concentration). (type N % calculated from total N content)**.**

C/O of GO	N-Doped Aerogel Synthesis Conditions	C/N	Pyridinic N %	Pyrrolic N %	Graphitic N %
2.159	85 °C, 1:2.5, 2 mg·mL^−1^	4.81	6.3	64.9	28.8
2.159	140 °C, 1:2.5, 2 mg·mL^−1^	6.32	0.0	81.5	18.5
1.85	140 °C, 1:2.5, 2 mg·mL^−1^	7.03	18.7	60	21.3
